# The Magnetic Properties of Fe/Cu Multilayered Nanowires: The Role of the Number of Fe Layers and Their Thickness

**DOI:** 10.3390/nano11102729

**Published:** 2021-10-15

**Authors:** Sofia Caspani, Suellen Moraes, David Navas, Mariana P. Proenca, Ricardo Magalhães, Cláudia Nunes, João Pedro Araújo, Célia T. Sousa

**Affiliations:** 1 IFIMUP and Departamento de Física e Astronomia, Faculdade de Ciências Universidade do Porto, Rua do Campo Alegre 687, 4169-007 Porto, Portugal; sofixsofi@gmail.com (S.C.); suellen.fisica@gmail.com (S.M.); mpproenca@fc.up.pt (M.P.P.); ricardo.magalhaes@fc.up.pt (R.M.); jearaujo@fc.up.pt (J.P.A.); 2 ICMM-CSIC-Instituto de Ciencia de Materiales de Madrid, Sor Juana Inés de la Cruz 3, 28049 Madrid, Spain; 3 ISOM and Dpto. Electrónica Física, Universidad Politécnica de Madrid, Avda. Complutense 30, 28040 Madrid, Spain; 4 LAQV, REQUIMTE, Faculty of Pharmacy of Porto University, 4050-313 Porto, Portugal; cdnunes@ff.up.pt

**Keywords:** nanowires, porous anodic alumina membranes, Fe/Cu bilayers, magnetization reversal

## Abstract

Multi-segmented bilayered Fe/Cu nanowires have been fabricated through the electrodeposition in porous anodic alumina membranes. We have assessed, with the support of micromagnetic simulations, the dependence of fabricated nanostructures’ magnetic properties either on the number of Fe/Cu bilayers or on the length of the magnetic layers, by fixing both the nonmagnetic segment length and the wire diameter. The magnetic reversal, in the segmented Fe nanowires (NWs) with a 300 nm length, occurs through the nucleation and propagation of a vortex domain wall (V-DW) from the extremities of each segment. By increasing the number of bilayers, the coercive field progressively increases due to the small magnetostatic coupling between Fe segments, but the coercivity found in an Fe continuous nanowire is not reached, since the interactions between layers is limited by the Cu separation. On the other hand, Fe segments 30 nm in length have exhibited a vortex configuration, with around 60% of the magnetization pointing parallel to the wires’ long axis, which is equivalent to an isolated Fe nanodisc. By increasing the Fe segment length, a magnetic reversal occurred through the nucleation and propagation of a V-DW from the extremities of each segment, similar to what happens in a long cylindrical Fe nanowire. The particular case of the Fe/Cu bilayered nanowires with Fe segments 20 nm in length revealed a magnetization oriented in opposite directions, forming a synthetic antiferromagnetic system with coercivity and remanence values close to zero.

## 1. Introduction

Considerable interest has arisen recently in studying 1D nanostructures, such as nanowires, nanopillars, and nanorods, owing to their potential applications [1,2]. The term nanowires (NWs) describes wires with a large length-to-diameter ratio, i.e., aspect ratio. Some of their remarkable properties arise from having a high density of electronic states, diameter-dependent band gaps, an enhanced surface scattering of electrons and photons, and high surface-to-volume ratios [3,4,5]. These properties lead to a unique electrical, optical, and magnetic behavior, making them suitable for many industrial and medical applications [2,6]. In addition, cylindrical NWs have been suggested as key elements for the development and understanding of a new research field known as magnetism in curved geometries [7]. It was recently demonstrated that the curved geometry of NWs can lead to novel and non-trivial magnetic phenomena, such as the formation of skyrmion magnetic configurations [8,9] and Bloch-point domain walls [10,11].

Among the several methods that can be employed to synthesize cylindrical NWs [12], template-assisted electrodeposition in porous anodic aluminum oxide (AAO) is one of the most reported techniques. The main advantages are its simplicity of controlling the size and shape of the nanostructures, without requiring expensive equipment or time-consuming processes [13,14]. Moreover, AAO templates allow for the fabrication of large areas of highly ordered nanostructures, which is particularly important when studying order-dependent properties such as magnetic responses.

The magnetic behavior of ferromagnetic NW arrays grown in AAO templates has been extensively investigated over the last two decades [15,16]. Besides single material-based NWs, multi-segmented NWs have gained increasing attention for applications in magnetic devices [17,18]. In particular, multilayered nanostructures, composed of several magnetic and non-magnetic materials, offer the possibility of restraining the magnetic interactions and tuning the magnetic anisotropic effects by changing the deposited material and/or the segments’ length [17,19].

The effect of the length of the magnetic layer on the magnetic behavior of cylindrical nanostructures has been largely investigated in Ni/Cu [17,20,21,22,23,24], Ni/Au [25], Co/Cu [26,27,28], CoFe/Au [29], CoFe/Cu [30] CoNi/Cu [31], and CoFeB/Cu [32] multilayered NWs. On the other hand, complementary studies have reported the magnetic behavior of multi-segmented NWs with tuned lengths of the non-magnetic layer, e.g., Co/Cu [27,33], Ni/Cu [21,23,34], CoFe/Au [29], CoFe/Cu [35] FeCoCu/Cu [36], FeCo/Cu [37], and CoFeB/Cu [32] nanostructures. These works have demonstrated that the magnetic behavior of the multilayered nanoarchitectures can be selectively tuned according to the required applications [20,38,39,40]. However, to employ such structures in biomedical applications, and since Co and Ni possess high toxicity levels, the need to fabricate completely biocompatible segmented NWs has arisen.

In this framework, few studies have been reported. Almasi-Kashi et al. [41] studied the magnetic properties of multi-segmented Fe/Cu NWs with very thin Cu spacer layers (<10 nm), corresponding to interacting magnetic layers. In a complementary experiment, Moraes et al. [19] investigated the role of the Cu segment’s length in multilayered Fe/Cu NWs. Their results showed that, for short Cu segments (<30 nm), the magnetic properties of the arrays were mainly controlled by the magnetostatic coupling between magnetic segments, behaving like a homogeneous array of Fe NWs. On the contrary, these nanoarchitectures behaved as an ensemble of non-interacting elements along the NW axis for longer non-magnetic spacers.

Therefore, in this work, we report the synthesis by template-assisted electrodeposition in porous AAO and the characterization of Fe/Cu multi-segmented NWs. We have investigated the changes in the magnetic behavior of multi-segmented Fe/Cu NWs resulting from varying the number of bilayers from 1 to 20, for fixed Fe and Cu lengths, or changing the length of the magnetic layer for two Cu layer lengths (60 and 120 nm). In addition, the Fe lengths have been substantially varied (from 20 to 345 nm) to allow for a better understanding of the magnetic properties of the nanostructures when modifying their aspect ratio.

## 2. Materials and Methods

Fe/Cu multi-segmented NWs were fabricated by electrochemical deposition from a single aqueous bath, using AAO templates as the working electrode. The AAO membranes were prepared from high-purity (>99.999%) Al foils by a standard two-step anodization process [16]. The first and second anodization steps lasted 24 and 48 h, respectively, having been performed at a constant voltage of 40 V in a 0.3 M oxalic acid solution, which was kept at a temperature of ≈2 °C. Through this process, self-organized AAO templates with a 1 cm diameter, a length of 120 µm, and pore diameter and interpore distances of *d* ≈ 35 ± 5 nm and *D_int_* ≈ 105 ± 5 nm, respectively, were obtained.

The Fe/Cu NWs were grown at room temperature by a DC pulsed electrodeposition method, as described in [19]. The used electrolyte contained 0.4 M H_3_BO_3_, 0.19 M FeSO_4_·7H_2_O, 0.005 M CuSO_4_·5H_2_O, and 0.003 M ascorbic acid (C_6_H_8_O_6_), and the electrodeposition was carried out by applying a −1.1 V versus Ag/AgCl reference electrode pulse to grow the Fe layer, followed by a pulse of −0.6 V to grow the Cu spacer.

To assess the morphological properties of the fabricated nanostructures, scanning electron microscopy (SEM) analysis has been performed by using an FEI Inspect F50 microscope (FEI Europe, Madrid, Spain). To investigate the structural properties of the wires, X-ray diffraction measurements in Bragg Brentano geometry have been executed through the use of a Rigaku SmartLab diffractometer (Rigaku Corporation, Tokio, Japan) with Cu-Kα radiation (1.540593 Å), 45 kV, and 200 mA. The magnetic hysteresis loops of Fe/Cu NW arrays have been measured with a vibrating sample magnetometer (VSM, LakeShore Controller, Model 7304) (LakeShore, Westerville, OH, USA). All measurements have been performed at room temperature, with the magnetic field applied parallel and perpendicular to the NWs’ long axis.

To better understand the magnetic results, 3-D micromagnetic simulations were also performed using the MuMax3 software (Version 3.9.1, DyNaMat group, Ghent University, Belgium) [42]. Due to the small amount of Cu contamination in the electrodeposited Fe segments, the magnetization of the Fe layers was set to *M*_Sat_ = 1600 emu/cm^3^, instead of the typical saturation magnetization value for Fe (1700 emu/cm^3^) [43]. While we used 0.5 as the damping parameter to ensure the rapid convergence of the simulations, the Fe exchange coupling constant and magnetocrystalline anisotropy values were fixed to *A* = 43 × 10^−8^ erg/cm [44,45] and *K* = 4.8 × 10^5^ erg/cm^3^, respectively. As the exchange length of our material is $l_{ex}=\sqrt{2A/{µ}_{0}M_{Sat}^{2}}\approx 5\;\mathrm{nm}$, the cell size was chosen to be (2.5 × 2.5 × 2.5) nm^3^.

## 3. Results and Discussion

### 3.1. Electrochemical Growth

**Figure 1:**
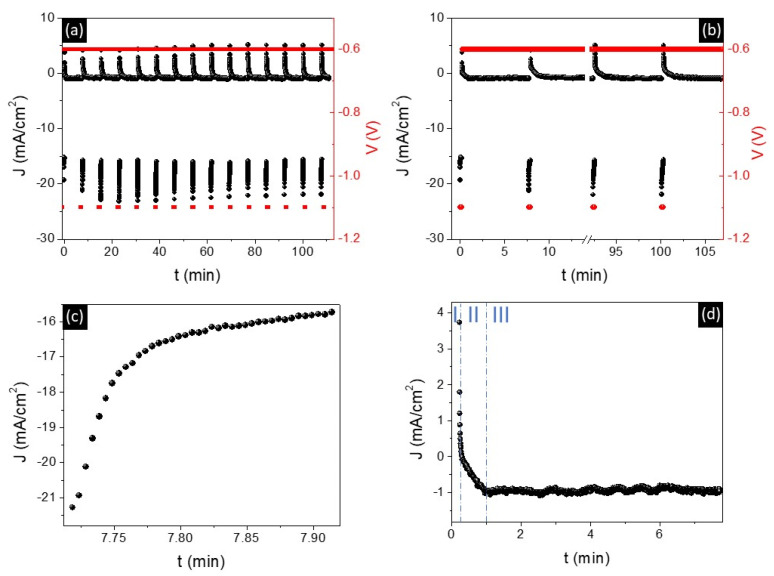
Current density transients of the pulsed electrodeposition process for the synthesis of the multi-segmented Fe/Cu nanowires (NWs) in anodic aluminum oxide (AAO) templates: (**a**) 15 Fe deposition pulses at −1.1 V and 15 Cu deposition pulses at −0.6 V. (**b**) Zoom of the first two and last two pulses of the Fe and Cu electrodeposition. Selected current density transients during the (**c**) Fe deposition and (**d**) Cu deposition processes.

A representative example of the potential sequence employed for the electrodeposition of multi-segmented Fe/Cu NWs is shown in Figure 1a,b. The possibility to perform a codeposition of Fe/Cu layers from a single bath is based on the difference between the reduction potentials of Cu (+0.14 V vs. Ag/AgCl electrode) and Fe (−0.644 V vs. Ag/AgCl electrode) and on the very low concentration of Cu ions in the deposition solution ([Cu^2+^] << [Fe^2+^]). These conditions ensure multilayers of Fe/Cu with low contamination since, for low Cu^2+^ concentrations, the Cu deposition is diffusion-limited over a wide potential range (from −0.15 to −0.7 V) [24,46,47]. Therefore, for an Fe deposition at high potentials (−1.1 V), the molar fraction of Cu in Fe layers is expected to be lower than 10%. Figure 1a shows the pulses required to grow 15 Cu/Fe bilayers and confirms the homogeneous growth of both layers.

The nucleation and growth mechanisms of the nanostructures, as well as their electrodeposition rates, can be determined using the current transients (Figure 1c,d). In general, the nucleation of each metal layer mainly depends on the nature of the previously electrodeposited layer and on the density of active sites on its surface, such as steps, kinks, or other defects, whereas its growth mechanism depends on the applied potential and electrolyte [47]. Depending on the nucleation rate, two limiting cases can be identified, i.e., instantaneous nucleation and progressive nucleation, which correspond to high and low nucleation rates, respectively [48,49]. In both cases, the current density starts to increase with time due to the 3D growth of the nucleation centers, which leads to an increase in the electrodeposition surface area. The current then achieves a quasi-constant value when the diffusion zones around the growing nuclei start to overlap, resulting in a 1D diffusion-limited current [48].

In the deposition of Fe layers (Figure 1c), a decrease in the absolute value of the current density is observed, which is associated with diffusion-limited growth. However, the expected initial increase in currents due to nucleation is not observed, due to the high deposition rate of Fe associated with the high deposition potential applied (−1.1 V). This fact indicates the occurrence of an instantaneous nucleation, where the nucleation sites become saturated after short times [50]. Regarding Cu, a more complex *J(t)* curve is observed (Figure 1d). In this case, the positive current observed in the first several seconds can be related to the oxidation of the previous Fe layer (Phase I). The absolute value of the current density transients then increases due to the Cu layer nucleation (Phase II), followed by a quasi-constant current density during the Cu layer growth (Phase III). Taking into account this type of curve, together with the quite low deposition potential of Cu (−0.6V) and the reduced concentration of Cu ions in the electrolyte, the Cu nucleation can be associated with a progressive nucleation behavior, where the density of nuclei increases linearly with time due to the low deposition rate [49,50].

Moreover, the total charge transients, *Q(t)*, led us to determine deposition rates of (0.322 ± 0.001) and (0.0037 ± 0.0003) nm/min for the Fe and Cu layers, respectively. The low deposition rate of Cu compared with Fe is mainly associated with the low concentration of Cu ions present in the solution. To explore the magnetic behavior of the segmented NWs as a function of the number of layers and the length of the Fe layers, different numbers of pulses and deposition times have been tested.

### 3.2. Morphological and Structural Characterization

**Figure 2:**
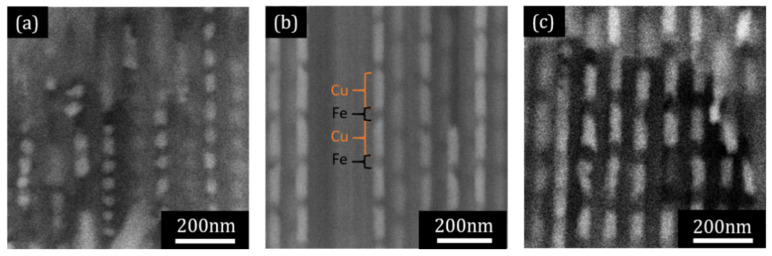
SEM cross-sectional views of multilayered Fe/Cu NWs embedded in AAO templates, exhibiting average Cu lengths of (**a**) 60 ± 7 nm and (**b**,**c**) 120 ± 5 nm and Fe lengths of (**a**) 20 ± 5 nm, (**b**) 30 ± 3 nm, and (**c**) 60 ± 8 nm.

Representative SEM cross-sectional images of the multilayered Fe/Cu NW arrays inside the AAO template are shown in Figure 2. The brighter and darker segments along the wires correspond to the Cu and Fe segments, respectively. Figure 2 illustrates representative NWs samples with highly homogeneous and well-defined segments, possessing Cu layer lengths of 60 and 120 nm, and Fe lengths varying between 20 and 60 nm.

X-ray diffraction (XRD) measurements (data not shown) of Fe NWs revealed a polycrystalline body-centered cubic (bcc) structure, with an intense peak corresponding to a preferential direction along the (110) crystallographic plane. The Cu layers presented a polycrystalline fcc structure, exhibiting a main peak corresponding to the (111) crystallographic plane.

### 3.3. Magnetic Properties

The resulting magnetic hysteresis loops of Fe/Cu NW arrays are presented and analyzed in the following sections, having been measured both in the parallel and perpendicular direction with respect to the wires’ long axis.

#### 3.3.1. Varing the Number of Bilayers

**Figure 3:**
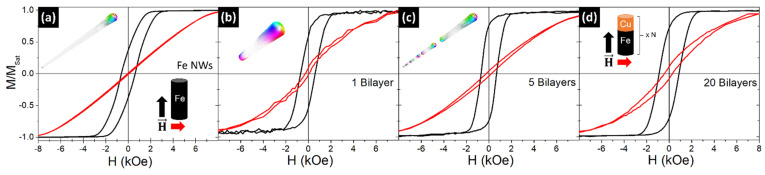
Magnetic hysteresis loops of (**a**) Fe NW arrays with 45 nm in diameter and 3000 nm in length and (**b**–**d**) Fe/Cu NW arrays with 45 nm in diameter, Fe lengths of ~300 nm, Cu segment lengths of ~120 nm, and (**b**) 1, (**c**) 5, and (**d**) 20 bilayers, measured along the parallel (black) and perpendicular (red) directions. Insets show the respective 3D simulated magnetic configurations at the switching field state, when applying the magnetic field parallel to the wire axis.

In this section, we assess the magnetic behavior of segmented NWs as a function of the bilayers’ number (*N*). The prepared samples consisted of *N* = 1, 3, 5, 15, and 20 bilayers, with Fe lengths of ~300 nm and Cu segments with a length of ~120 nm. Previous works have shown that, when increasing the length of the non-magnetic layer above the wire diameter value, the interactions between the magnetic layers along the wire become negligible and the system behaves as an ensemble of non-interacting entities [19,51]. The magnetic hysteresis loops for an Fe NW array (3 µm in length) and the Fe/Cu NWs with 1, 5, and 20 bilayers are presented in Figure 3. In all cases, an increase in the coercivity and remanence values is observed when the magnetic field is applied parallel to the wire’s axis, confirming the anisotropic behavior of the structures. A similar magnetic behavior has been observed for all samples, being independent of the number of bilayers.

To analyze the magnetization reversal modes in Fe/Cu NWs as a function of the number of bilayers, 3-D micromagnetic simulations using the MuMax3 software (Version 3.9.1) [42] were performed. Following the experimental results and our previous work [19], multi-segmented individual NWs 40 nm in diameter, with ferromagnetic Fe layers 300 nm in length and non-magnetic Cu spacers 120 nm in length, were simulated. The number of bilayers was varied from 1 to 15. An individual long Fe NW (3 µm in length) was also simulated for comparison (inset in Figure 3a).

In agreement with the literature [52], micromagnetic simulations show that the magnetization reversal process in long cylindrical Fe NWs (3 µm in length) occurs through the nucleation and propagation of a vortex domain wall (V-DW) from the NW extremities (see inset in Figure 3a). Similarly, the magnetic reversal in the 300 nm length segmented Fe NWs occurs through the nucleation and propagation of a V-DW from the extremities of each segment, regardless of the number of bilayers (see insets in Figure 3b,c).

**Figure 4:**
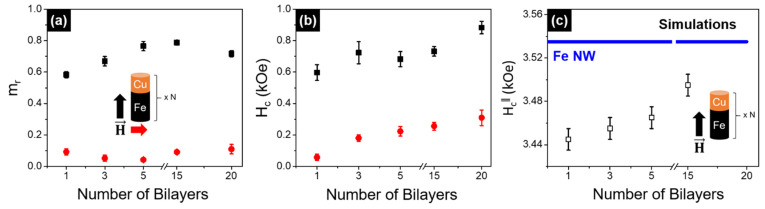
(**a**) Normalized remanence (*m_r_*) and (**b**) coercivity (*H_c_*) values as a function of the number of Fe/Cu bilayers, measured when applying a magnetic field parallel (black squares) and perpendicular (red dots) to the wire’s axis. (**c**) Coercive field of the segmented Fe/Cu NWs, as a function of the number of layers (open symbols), and of the 3 µm length isolated Fe NW (blue continuous line) extracted from the simulated hysteresis loops when the external field was applied parallel to the NW axis.

Figure 4a,b presents the normalized remanence and coercivity values, respectively, as a function of the number of Fe/Cu bilayers. The coercivity values were found to slightly increase with the number of bilayers, either when the field is applied parallel or perpendicular to the wire’s axis. On the other hand, the remanence values are almost constant along the perpendicular direction, while they seem to first increase with the number of bilayers (up to 5 bilayers) and then keep constant for longer NWs (5–20 bilayers). This phenomenon can be associated with the complex behavior of the magnetization reversal mechanism in multilayered structures, in which interactions between neighboring NWs must be considered [17,19].

From the simulated hysteresis loops of the isolated Fe/Cu NWs with the magnetic field applied along the wire axis, we have extracted the coercivity fields (*H_C_*) as a function of the number of bilayers (open symbols in Figure 4c) and for the long Fe NWs (continuous blue line in Figure 4c). Despite the significant difference between the *H_C_* values obtained experimentally and the simulated ones, a good correlation with the experimental data was achieved (Figure 4b,c). Low-aspect-ratio segmented NWs with 35-nm-length Fe segments present a behavior almost like a set of non-interacting nanoparticles when they are separated by 120-nm-length non-magnetic Cu spacers [19]. However, when we increase the Fe length to 300 nm, the contribution of the magnetostatic interactions is not negligible, as shown in Figure 3 and Figure 4. The coercive field progressively increases with the number of bilayers due to the small magnetostatic coupling between Fe segments, but the maximum value obtained does not reach the *H_C_* of the continuous Fe NW, since the interaction between layers is limited by the Cu separation.

#### 3.3.2. Varying the Fe Length

After analyzing the magnetic behavior of Fe/Cu NWs as a function of the number of bilayers, in this section we focus our attention on understanding the influence of the length of the Fe layer on the interactions between the magnetic layers. For that purpose, we have prepared multiple Fe/Cu NWs’ samples, where the number of bilayers was fixed to 15, and the Fe segments’ length was varied between 20 and 300 nm. In addition, the effect of the non-magnetic spacer length has been explored using Cu segments with lengths of 60 and 120 nm.

**Figure 5:**
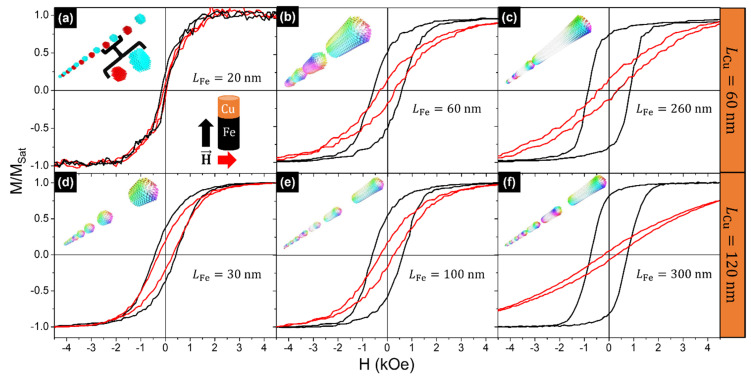
Magnetic hysteresis loops of multi-segmented Fe/Cu NW arrays with different Fe segment lengths (*L*_Fe_) and Cu segment lengths (*L*_Cu_) of (**a**–**c**) 60 nm and (**d**–**f**) 120 nm, measured along the parallel (black) and perpendicular (red) directions. Insets show the respective 3D simulated magnetic configurations at the switching field state, when applying the magnetic field parallel to the wire axis.

Figure 5 presents the magnetic hysteresis loops of the Fe/Cu NW samples with 15 bilayers. As previously reported [53], the magnetic anisotropy in large-aspect-ratio NWs is dominated by the shape anisotropy contribution, leading to the alignment of the easy magnetization axis along the NWs’ longitudinal axis (Figure 5b,c,e,f). Regarding the coercivity and remanence values along the parallel direction, a significant increase of both parameters is observed for longer Fe segments, which is accompanied by an increase in the saturation field along the perpendicular direction.

On the other hand, the hysteresis loops of the NWs with the smallest Fe segments (20–30 nm), which correspond to aspect ratios <1, show that the easy magnetization axis deviates from the longitudinal direction and suggests an isotropic-like behavior (see Figure 5a,d), in agreement with previous studies [19,54]. In addition, a significant difference in the hysteresis loops can be observed when the Cu segment length is increased from 60 to 120 nm. The easy magnetization axis of Fe/Cu NWs with 20-nm-length Fe segments and 60-nm-length Cu spacers tends to be perpendicular to the longitudinal wire axis, and the system behaves like a synthetic antiferromagnet, presenting negligible coercivity and remanence values (Figure 5a). On the contrary, and as will be further confirmed by the micromagnetic simulations described below, the easy magnetization axis deviates from the perpendicular direction towards a more parallel one for Fe/Cu NWs with Fe and Cu segment lengths of 30 nm and 120 nm, respectively.

To confirm that the NWs exhibit different magnetization reversal regimes as a function of the Fe segment aspect ratio, the study was complemented by performing 3-D micromagnetic simulations (MuMax3 software, Version 3.9.1) [42]. In this case, we have simulated multi-segmented individual NWs 40 nm in diameter, varying the Fe layer length from 20 to 300 nm, considering two different lengths for the non-magnetic Cu spacers (60 and 120 nm) and keeping the total number of bilayers fixed at 15.

The micromagnetic simulations showed that the segmented Fe/Cu NWs behaved like a set of 15 non-interacting nanoparticles when the Fe and Cu spacer lengths were 30 and 120 nm, respectively (see inset in Figure 5d). In addition, it was confirmed that the 30-nm-length Fe segments (separated by 120 nm of Cu) exhibited a vortex configuration with around 60% of the magnetization pointing parallel to the NW long axis. As soon as the Fe segment lengths were increased (≥100 nm), while keeping the Cu segments to 120 nm, the magnetic reversal mode occurred through the nucleation and propagation of a V-DW from the extremities of each segment (see insets in Figure 5e,f), similar to what happened in the longer cylindrical Fe NW (inset in Figure 3a). This behavior becomes more evident as the Fe segments’ length is increased.

To study the effect of the non-magnetic Cu spacer layer, Fe/Cu NWs with Cu spacers 60 nm in length and Fe layers with lengths ranging from 20 to 260 nm were also simulated. The 3D simulated magnetic configuration at remanence of the Fe/Cu NWs with Fe segments 20 nm in length showed an easy magnetization axis lying perpendicular to the longitudinal NW’s axis (inset in Figure 5a). In addition, the magnetization in consecutive Fe segments is oriented in opposite directions, confirming the formation of a synthetic antiferromagnetic system with coercivity and remanence values close to zero (Figure 5a). As was observed in the samples with Cu spacer lengths of 120 nm, the magnetization reversal evolved from an in-plane (perpendicular) configuration to the nucleation and propagation of a V-DW from the extremities of each segment for NWs with longer Fe segments (≥60 nm).

**Figure 6:**
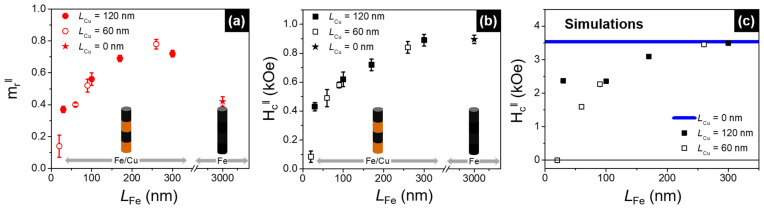
(**a**) Reduced remanence and (**b**) coercivity values as a function of the Fe length, measured when applying a magnetic field parallel to the wires’ long axis. (**c**) Coercive field as a function of the Fe segment length in Fe/Cu NWs with a Cu spacer length of 120 (full symbols) and 60 nm (open symbols), as well as in the 3-µm-length isolated Fe NW (blue continuous line), extracted from the simulated hysteresis loops when the external field was applied parallel to the NW’s longitudinal axis.

Table 1 summarizes the results obtained, including the lengths of the Fe segments together with the coercivity and normalized remanence values measured along both the parallel and perpendicular directions of the applied field. In addition, the coercivity and reduced remanence values are also presented in Figure 6, as a function of the Fe segments’ length, considering the external magnetic field applied parallel to the NWs’ long axis. Both the coercivity and remanence values were found to progressively increase with increasing Fe length in the multi-segmented Fe/Cu NWs. However, while the parallel coercivity increased until the value corresponding to the long Fe NW was reached (Figure 6b), the remanence values reached even higher values when compared to the continuous Fe NW (Figure 6a). This may be ascribed to the stronger magnetostatic interactions between neighboring wires for the long Fe NWs when compared to multi-segmented Fe/Cu NWs, which decrease the respective remanence values [55].

Regarding the evolution of the simulated coercive fields as a function of the Fe segment length for Cu spacer lengths of 60 and 120 nm (Figure 6c), these values progressively increased with the Fe length, approaching the value corresponding to the long Fe NW. Again, and despite having simulated only one wire, a good qualitative correlation with the experimental data was achieved, demonstrating that larger magnetostatic interactions are acting on the NWs when the Fe segments are longer.

## 4. Conclusions

In this work, bi-segmented multilayered Fe/Cu NWs have been successfully fabricated by pulsed electrodeposition in AAO templates, presenting a diameter of 45 nm and variable aspect ratios. Their morphological characterization revealed uniform and distinguishable layers, while the structural one showed a polycrystalline body-centered cubic (bcc) structure for either the Fe or Cu NWs. The magnetic measurements and micromagnetic simulations have demonstrated that the behavior of the Fe/Cu NWs can be easily tuned by increasing the number of layers and/or the ferromagnetic layer lengths. In this context, it was shown that the easy magnetization axis is mainly ruled by the shape anisotropy contribution. It was observed that the coercivity field values vary with the number of segments. Therefore, the required *H_C_* can be selectively obtained by controlling the number of bilayers, making it possible to achieve values ranging between the coercivity of a segmented NW with only one segment and the value observed for an Fe NW with a length of several micrometers.

Furthermore, the magnetic configuration at remanence can range from a single domain parallel to the longitudinal wire axis, for Fe lengths ≥60 nm, to a vortex configuration, for NWs with Fe segments exhibiting lengths of ≈30 nm. For layered NWs with Fe segments presenting 20 nm in length, the easy magnetization axis lies in the sample plane or perpendicular to the longitudinal wire axis, illustrating a synthetic antiferromagnet behavior with negligible coercivity and remanence values.

In summary, it was demonstrated that multilayered NWs can be fabricated with a well-controlled magnetic behavior. We believe that this possibility of achieving fine tuning and control of the magnetic response of multilayered NWs may be of great interest in technological applications that require high sensitivity and selectivity, e.g., for the development of magnetic sensor devices.

## Figures and Tables

**Table 1:** Magnetic properties of multi-segmented NWs: Coercive field ($H_{c}$) and normalized remanence ($m_{r}$) measured with the magnetic field applied parallel (∥) and perpendicular (⊥) to the NWs’ longitudinal axis.

Systems	$L_{Fe}\;(\mathrm{nm})$	$H_{c}^{\|}\;(\mathrm{Oe})$	$H_{c}^{⊥}\;(\mathrm{Oe})$	$m_{r}^{\|}\;$	$m_{r}^{⊥}\;$
${\left({\mathrm{Fe}}_{\left(20\;\mathrm{nm}\right)}/{\mathrm{Cu}}_{\left(60\;\mathrm{nm}\right)}\right)}_{15}$	20 ± 5	84 ± 40	60 ± 40	0.15 ± 0.04	0.09 ± 0.01
${\left({\mathrm{Fe}}_{\left(60\;\mathrm{nm}\right)}/{\mathrm{Cu}}_{\left(60\;\mathrm{nm}\right)}\right)}_{15}$	60 ± 7	490 ± 60	350 ± 30	0.40 ± 0.10	0.25 ± 0.05
${\left({\mathrm{Fe}}_{\left(260\;\mathrm{nm}\right)}/{\mathrm{Cu}}_{\left(60\;\mathrm{nm}\right)}\right)}_{15}$	260 ± 26	840 ± 40	390 ± 100	0.78 ± 0.03	0.11 ± 0.08
${\left({\mathrm{Fe}}_{\left(30\;\mathrm{nm}\right)}/{\mathrm{Cu}}_{\left(120\;\mathrm{nm}\right)}\right)}_{15}$	30 ± 3	430 ± 30	280 ± 35	0.37 ± 0.02	0.20 ± 0.02
${\left({\mathrm{Fe}}_{\left(100\;\mathrm{nm}\right)}/{\mathrm{Cu}}_{\left(120\;\mathrm{nm}\right)}\right)}_{15}$	100 ± 8	620 ± 45	260 ± 50	0.56 ± 0.04	0.17 ± 0.03
${\left({\mathrm{Fe}}_{\left(300\;\mathrm{nm}\right)}/{\mathrm{Cu}}_{\left(120\;\mathrm{nm}\right)}\right)}_{15}$	300 ± 60	890 ± 40	363 ± 70	0.72 ± 0.02	0.09 ± 0.03
